# The role of brain MR and FDG-PET in the diagnosis of neurodegenerative disease

**DOI:** 10.1007/s00330-025-11940-3

**Published:** 2025-09-03

**Authors:** Yoshimi Anzai, Satoshi Minoshima

**Affiliations:** Department of Radiology and Imaging Sciences, University of Utah, 30N Mario Capecchi Drive, 2 South, Salt Lake City, UT USA

**Keywords:** Brain, Magnetic resonance imaging, Neurodegenerative disease, Dementia

## Abstract

**Abstract:**

Alzheimer disease (AD) is the most common dementing disorder, affecting 55 million people worldwide. Brain MRI plays an integral role in the diagnostic evaluation of patients with cognitive symptoms. When interpreting brain MRI for cognitive impairment, radiologists should assess the following four key features: (1) white matter ischemic burden, (2) structural changes to suggest normal pressure hydrocephalus, (3) locoregional pattern of brain atrophy, and (4) presence of microhemorrhage or superficial siderosis, particularly for determining eligibility for anti-amyloid monoclonal antibody (MAB) treatment when appropriate. The recent approval and clinical adoption of anti-amyloid MAB expanded the role of neuroradiologists in evaluating eligibility and monitoring ARIA (amyloid-related imaging abnormality) among patients receiving anti-amyloid MAB. This advancement underscores the importance of standardized imaging protocols and effective communication between neuroradiologists and cognitive neurologists. Depending on the severity of ARIA and patients’ symptoms, treatment may need to be suspended or discontinued. This review article explores brain MRI and FDG-PET/CT imaging abnormalities in patients with major cognitive and movement disorders associated with dementia. It aims to assist radiologists in providing differential diagnoses within a clinical context. Finally, the article emphasizes the importance of recognizing co-pathologies, since patients may have more than one neurodegenerative disease rather than viewing these neurodegenerative diseases as being mutually exclusive.

**Key Points:**

***Question**** Traditional regional patterns of brain atrophy on MRI by neuroradiologists may not be effective given the recent advances in understanding of neurodegenerative disease and recognition of co-pathologies*.

***Findings**** The locoregional atrophy and the patterns of metabolic abnormality help in the differential diagnosis of neurodegenerative disease. Remember that brain MRI determines eligibility for anti-amyloid immunotherapy*.

***Clinical relevance**** Understanding clinical history is vital for interpreting brain MRI for patients with cognitive impairment or memory loss. Newly recognized entities such as limbic-predominant age-related TDP43 encephalopathy (LATE) can mimic Alzheimer disease among extremely elderly patients with amnestic symptoms with mesial temporal lobe atrophy*.

## Introduction

Neurodegenerative disease is a devastating disease for patients, families, communities, and healthcare systems. There were 55 million people suffering from dementia worldwide [1] in 2020. The number is expected to double every 20 years, reaching 78 million by 2030, and 139 million in 2050 (https://www.alzint.org/about/dementia-facts-figures/dementia-statistics/). The most common dementing neurodegenerative disease is Alzheimer’s disease. The percentage of people with AD increases with age. As the population over 65 is projected to grow due to global longevity, the number and proportion of Americans with AD or other dementing disorders are expected to increase [2]. Almost two-thirds of Americans with AD are women, although part of the difference might be explained by the fact that women tend to live longer than men.

The diagnostic workup of patients with cognitive impairment includes laboratory tests (e.g., thyroid function), vitamin B12, physical examinations, movement testing, and neurocognitive testing. Structural brain imaging, such as brain MRI, is an essential part of the clinical workup for patients with memory loss, cognitive decline, or dementia.

In this review article, we will discuss the basic brain MRI features for patients with neurodegenerative diseases (Table 1), in particular, (1) Imaging Sequences, (2) Imaging assessment for patients suspected of cognitive impairment, and (3) Specific patterns of atrophy for common neurodegenerative diseases. Brain MRI also excludes structural diseases that mimic neurodegenerative diseases. Once the neurodegenerative disease is likely to explain the patient’s clinical symptoms, FDG-PET CT is frequently used for differential diagnoses of various dementing disorders. In contrast, amyloid PET is to detect the presence of amyloid pathology that leads to the diagnosis of Alzheimer disease and supports a patient’s eligibility for anti-amyloid treatment. This review also includes various clinical cases where brain MRI and FDG-PET/CT are used complementarily to make a specific diagnosis of dementing disorder and guide clinicians in the management of patients. Advances in molecular imaging or functional brain MRI are beyond the scope of this review.

**Table 1:** Neurodegenerative disease, underlying proteinopathy, and MR imaging findings

	Proteinopathy	Brain MRI findings
AD	Amyloid A beta 42, Tauo (4R and 3R)	Parieto-posterior temporal atrophy, mesial temporal atrophy
FTLD	Tauo, TDP-43	Frontal atrophy for bvFTD, Anterior temporal atrophy for svPPA, Left posterior frontal and insular atrophy for nfPPA, parietal and posterior temporal atrophy for lpPPA
DLB	Alpha-Synuclein, low level of Amyloid	Non-specific in early disease, mild parietal and occipital atrophy in late phase
PD	Alpha-Synuclein	Loss of high signal of nigrosome 1 in substantia nigra on SWI and increased iron deposition in the dorsolateral putamen
PSP	4R Tau	Atrophy of midbrain tegmentum, superior cerebellar peduncle
CBD	4R Tau	Atophy of midbrain tegmentum, assymmetric atrophy of posterior frontal and parietal gray matter with white matter T2 hyperintensity
MSA	Alpha-Synuclein	Depends on subtypes, Hott cross bun sign for cMSA, increased iron deposition along the dorsolateral putamen in pMSA
LATE	TDP-43	Profound atrophy of hippocampus, amygdala, and mild atrophy in the middle frontal gyrus

*AD* Alzheimer disease, *CBD* corticobasal degeneration, *DLB* dementia with Lewy body, *FTLD* frontotemporal lobar degeneration, *LATE* limbic-predominant age-related TDP43 encephalopathy, *MSA* Multiple system atrophy, *PD* Parkinson disease, *PSP* progressive supranuclear palsy, *TDP 43* transactive response DNA-binding protein of 43 kDa

## Imaging techniques

### Brain MR technique

Brain MR imaging techniques include the basic T2-weighted axial images, T2 FLAIR axial images, diffusion-weighted images with ADC (Apparent Diffusion Coefficient), Gradient Echo or SWI (susceptibility-weighted) imaging, and the 3D high-resolution T1-weighted sequences such as MPRAGE (magnetization prepared rapid gradient echo) with a 1–1.2 mm slice thickness for quantitative volumetric analysis. Various quantitative analysis software is available and FDA-approved. The high-resolution 3D T1-weighted sequences allow the quantitative assessment of brain segmentation and volume compared to the age-matched controls, and serve as a supplement to visual assessment or regional volume loss assessment by radiologists.

### FDG-PET/CT technique

FDG-PET/CT of the brain is performed following 6 h of fasting. Approximately 60 min after intravenous administration of 18F-Fluorodeoxyglucose, images were obtained from the skull base to the vertex of the head. In addition to axial, coronal, and sagittal images of FDG-PET and corresponding CT images, statistical mapping, such as 3D-SSP (stereotactic surface projection) images of the FDG-PET/CT, can significantly improve the diagnostic accuracy. The 3D-SSP allows visualization of statistically abnormal metabolic reduction in comparison to the normal database [3, 4].

## Imaging assessment of neurodegenerative disease

When interpreting brain MRI for patients with cognitive impairment, 4 specific items to be included in the reports: (1) the extent of microvascular ischemic disease (MVID), (2) the pattern of sulcal or ventricular dilatation, (3) the pattern of regional brain atrophy, and (4) the presence of microhemorrhage or superficial siderosis to suggest cerebral amyloid angiopathy (CAA).

1. The extent of white matter T2/FLAIR signal abnormality—often called white matter hyperintensities (WMH), small vessel disease (SVD), or microvascular ischemic disease (MVID) is often associated with cognitive decline [5]. Patients with severe WMH often have vascular risk factors such as hypertension, hyperlipidemia, diabetes, a history of stroke, and smoking. The Meta VCI Map consortium study from 9 cohorts showed a dose-dependent inverse relationship between WMH volume and post-stroke cognitive function among post-stroke patients [6]. The underlying etiology includes endothelial dysfunction, blood-brain barrier integrity, and inflammation. The degree of WMH can be qualitatively assessed using the Fazekas scale [7], which has been widely adapted over decades. A prospective study showed that WMH and WM T1 hypointensity volume correlate with CSF beta-amyloid in non-demented elderly subjects [8].
2. The pattern of sulcal and ventricular dilatation. Normal pressure hydrocephalus (NPH) is often seen in elderly patients who present with cognitive decline, incontinence, and lower extremity weakness, which may result in frequent falls. The classic imaging findings include the effacement of parasagittal frontoparietal cortical sulci, dilatation of Sylvian fissures, and a narrowed callosal angle (measured at the level of the posterior commissure) [9]. The imaging findings are also referred to as disproportionately enlarged subarachnoid space hydrocephalus (DESH) [10]. DESH is imaging patterns often seen in the setting of NPH. If no clinical symptoms of NPH are present, it is referred to as DESH, rather than NPH. Improvement of neurological symptoms after high-volume lumbar puncture predicts a favorable response to lumbar shunt.
3. The pattern of regional atrophy. Under healthy aging conditions, the brain is expected to reduce volume at 0.5–1% per year [11]. In patients with dementia, volume loss is accelerated compared to healthy subjects. In addition, regional volume loss is seen in specific dementing disorders. In other words, what part of the brain shows more profound atrophy is one of the diagnostic clues for making a specific neurodegenerative disease. The pattern of atrophy may suggest a specific diagnosis when present in conjunction with the clinical presentation. Since atrophy (neuronal loss) is a downstream phenomenon in neurodegenerative disease, structural brain volume loss does not help detect early neurodegenerative disease. Various qualitative assessment classifications, such as MTA (medial temporal atrophy) or GCA (global cortical atrophy) scores, were developed and widely adopted [12–15]. The scoring system allows systematic data collection and guidance on the severity of atrophy, although it is subject to inter-observer variability. Quantitative volumetric analysis software mitigates the inter-observer variability and offers quantitative measurements and percentages for age-matched cohorts. Although some software is FDA-approved, the accuracy of these quantitative assessments in the real-world setting remains to be determined.
4. Cerebral amyloid angiopathy (CAA) is an age-related vascular disease in which amyloid-beta (Aβ) deposition affects the cortical and leptomeningeal vascular walls. CAA causes lobar intraparenchymal hemorrhage and superficial siderosis in the elderly and is an independent risk factor for age-related cognitive impairment. Although amyloid plaques in AD consist mostly of Aβ 42, Aβ 40, as seen in CAA, is several times more abundant than Aβ 42. Due to the recent clinical application of anti-amyloid therapy, the assessment is CAA is critical to determine the eligibility of patients for anti-amyloid treatment. The clinical trials often exclude patients with more than 5 microhemorrhages and any superficial siderosis from entering anti-amyloid treatment. Please note that MAB therapy is currently not approved for patients with APOE ε4 homozygotes in British and European authorities due to its high risk of ARIA (Amyloid-related imaging abnormality) development. Please note that the sensitivity of detecting microhemorrhage or superficial siderosis depends on the MR sequence, i.e., GRE versus SWI and the magnetic field strength. SWI sequence is reported to be more sensitive in detecting microhemorrhages. It is still debatable which sequence to use. Under the current guidance, the GRE is recommended as it is widely available at community hospitals.

Another imaging feature that is on the topic of interest is perivascular space (PVS) and choroid plexus, since they reflect indirect signs of impaired glymphatic function– especially interstitial fluid exchange to remove brain waste products such as amyloid or tau. PVS increases with aging, just like WM hyperintensity. A large study of multiple normative population-based data provided a visual grading system of PVS at the basal ganglia and white matter based on patients’ age [16]. Dilated PVS is also considered a part of the indirect sign of impaired glymphatic function and is often associated with cognitive impairment and sleep disorders [17]. Dilated PVS independently predicts amyloid positivity [18]. Although there is a strong suggestion that dilated PVS is a sign of impaired glymphatic system, the findings remain non-specific and can be seen in the setting of idiopathic intracranial hypertension or impaired CSF fluid dynamics [19, 20]. Remember that the glymphatic function is a dynamic process known to decrease with a lack of sleep among healthy subjects, even for 1 day [21]. The development and validation of non-invasive, repeatable measures to address glymphatic function facilitate the investigation of various behavioral modification therapies for neurodegenerative disorders.

### Anti-amyloid treatment and amyloid-related imaging abnormalities

Anti-amyloid monoclonal antibody (MAB) therapy targets the amyloid-beta (Aβ) protein and reduces Aβ levels in the brain, potentially serving as a disease-modifying therapy for patients with mild cognitive impairment and early Alzheimer disease. Increasing clinical use of anti-amyloid MAB therapy led to an additional responsibility for neuroradiologists, which is the determination of the eligibility and detection of ARIA that potentially led to the temporal or permanent discontinuation of MAB, depending on clinical symptoms and severity of ARIA [22].

The consistent adoption of the standardized MR protocol in the AJNR White Paper [23] is highly recommended. ARIA has two distinct features: AIRA-E (edema and effusion) and ARIA-H (microhemorrhage and superficial siderosis) [24]. The size of ARIA-E and the number of new ARIA-H determine the radiographic severity of ARIA. The detailed mechanism of ARIA development is beyond the scope of this review.

## Neurodegenerative disease

For the sake of simplicity, we will discuss imaging features of neurodegenerative diseases as if each disease were an independent disease, such as Alzheimer’s disease (AD), Frontotemporal lobar degeneration (FTLD), and dementia with Lewy body (DLB). However, the presence of co-pathologies has been increasingly recognized recently, as patients may have features of more than one neurodegenerative disease. Much of the Brain MR diagnosis of dementia depends on the pattern of brain atrophy and neuronal loss, manifesting as widening of cortical sulci and adjacent ventricles or cortical thinning. However, neuronal loss is a late manifestation of the neurodegenerative disease; thus, detecting atrophy is not a sensitive marker for early diagnosis. It is well known that cerebral amyloid deposition can be seen even 20 years before patients may become symptomatic, however patients might never suffer from dementia in their lives [25].

### Imaging appearance of neurodegenerative diseases (NDD)

Recent advancements in understanding have revealed that neurodegenerative disease (NDD) is a highly complex disease entity with various overlapping features among subgroups of NDD. The traditional classification of AD, DLB (Dementia with Lewy Bodies), and FTD (Frontotemporal dementia) as if they were mutually exclusive entities is no longer widely accepted [26]. Underlying proteinopathy, in addition to clinical phenotypes, better characterizes NDD. Furthermore, new entities are increasingly reported, including LATE (limbic-predominant age-related TDP-43 encephalopathy) [26, 27] or FUS (fused in sarcoma), which makes pattern recognition by imaging phenotypes or clinical manifestations challenging, if not impossible.

### Alzheimer disease (AD)

Alzheimer disease is named after Dr. Alois Alzheimer, a German pathologist, who examined the brain of a woman who had died after suffering from memory loss, language problems, and unpredictable behavior. He found many clumps (plaques) and tangled bundles (neurofibrillary tangles) in her brain. AD is ranked as the seventh leading cause of death in the US and the most common cause of dementia among older adults.

**Fig. 1:**
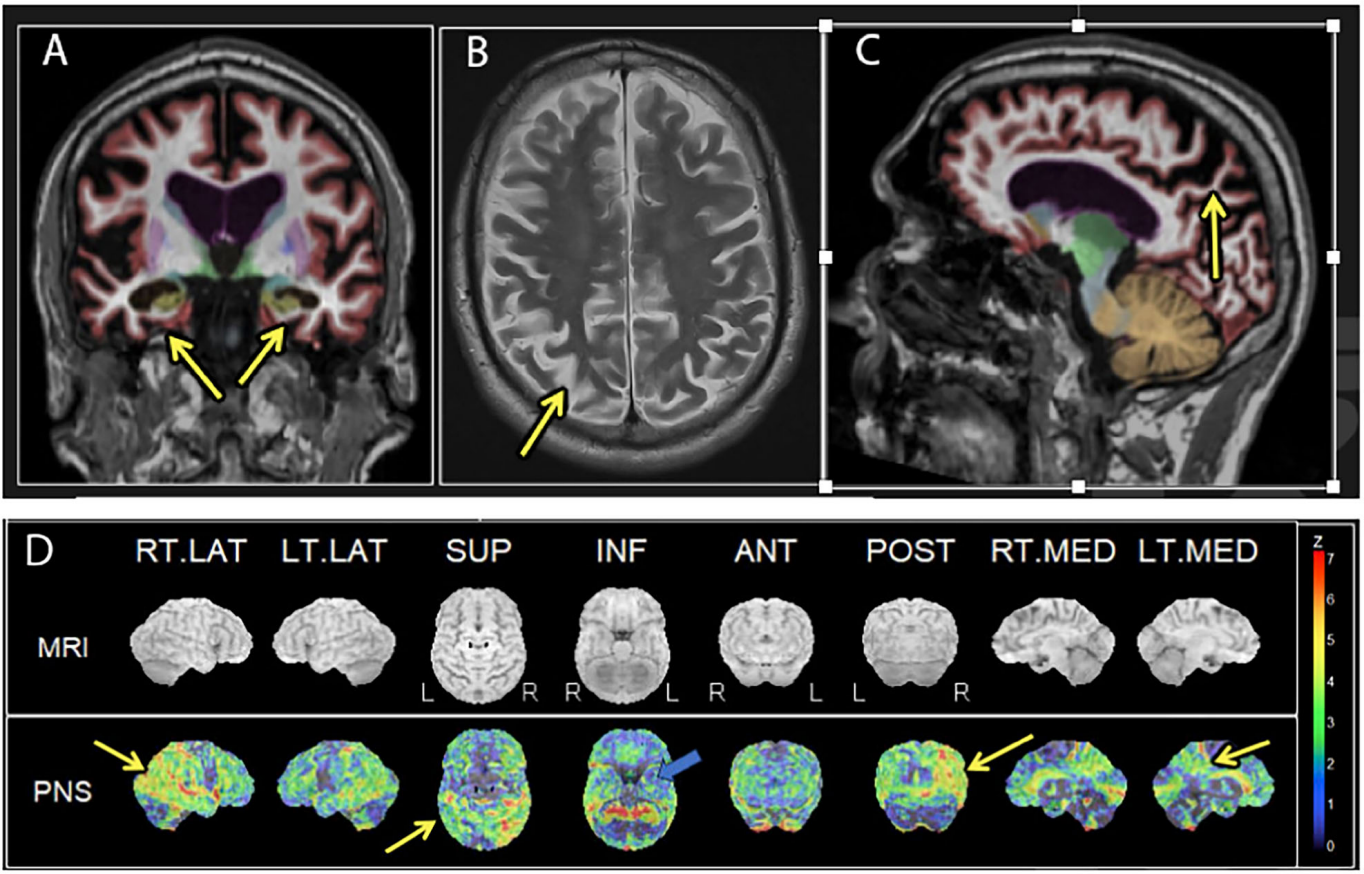
76-year-old male with severe progressive memory loss and cognitive dysfunction. T1-weighted coronal (**A**), T2-weighted axial (**B**), and T1-weighted sagittal (**C**) images of brain MR show parietal and medial temporal volume loss. FDG-PET with 3D-SSP (**D**) demonstrates profound posterior cingulate, parietal and posterior temporal metabolic reduction, right greater than left (yellow arrows). Hippocampal hypometabolism is mild (blue arrow). The findings are consistent with Alzheimer disease

The typical brain MR imaging findings of AD include parietal and posterior temporal volume loss and medial temporal lobe atrophy (Fig. 1). However, brain MRI may appear normal in the early stage of AD, or the atrophy pattern might be seen in patients without AD.

A recent systematic review and meta-analysis reveals heterogeneity of AD with 4 biological subtypes of AD [28]: typical, limbic-predominant, hippocampal-sparing, and minimal atrophy AD. Typical AD shows atrophy in both the hippocampus and the association cortex. The hippocampal sparing affects younger age at presentation and is the most aggressive form with faster progression, whereas limbic-predominant AD affects elderly patients and slow progression, probably due to the contribution from TDP-43 pathology [29]. APOE ε4 carriers are more frequently found in the typical AD and limbic-predominant AD. Hippocampal-sparing AD has the highest level of education (higher cognitive reserve), and minimal atrophy AD has the lowest level of education. The exact reason for the relationship between the level of education and hippocampal atrophy is unknown. Based on FDG-PET, hippocampal-sparing AD reveals greater hypometabolism in the association cortex. A postmortem study shows increased Lewy body pathology in hippocampal-sparing AD [30].

Atypical nonamnestic presentations of AD variants, such as PCA (posterior cortical atrophy- visual variant of AD), logopenic variant PPA, and frontal variant of AD, are more common in hippocampal sparing than in typical and limbic predominant AD.

Another classification of AD is based on the age of onset, dividing it into an early onset AD (EOAD) and late onset AD (LOAD), which are arbitrarily defined by age of onset before or after 65 years old. EOAD is rare (5–10%) and is often associated with genetic predisposition (Presenilin 1 and 2, and Amyloid Precursor Protein). Additionally, 35–60% of EOADs have at least one affected first-degree relative with AD. EOAD presents with a more aggressive and non-amnesic type, sparing the hippocampus [31, 32]. A wider range of environmental and lifestyle factors influences LOAD. ApoE is a well-established risk factor for LOAD, but not all carriers of the ApoE allele 4 develop LOAD. Quantitative MR assessments of EOAD and LOAD revealed greater parietal lobe atrophy in EOAD than in LOAD [33]. FDG-PET imaging findings of AD include metabolic reduction in the posterior cingulate and precuneus, and parietal and posterior temporal lobes (Fig. 1D).

**Fig. 2:**
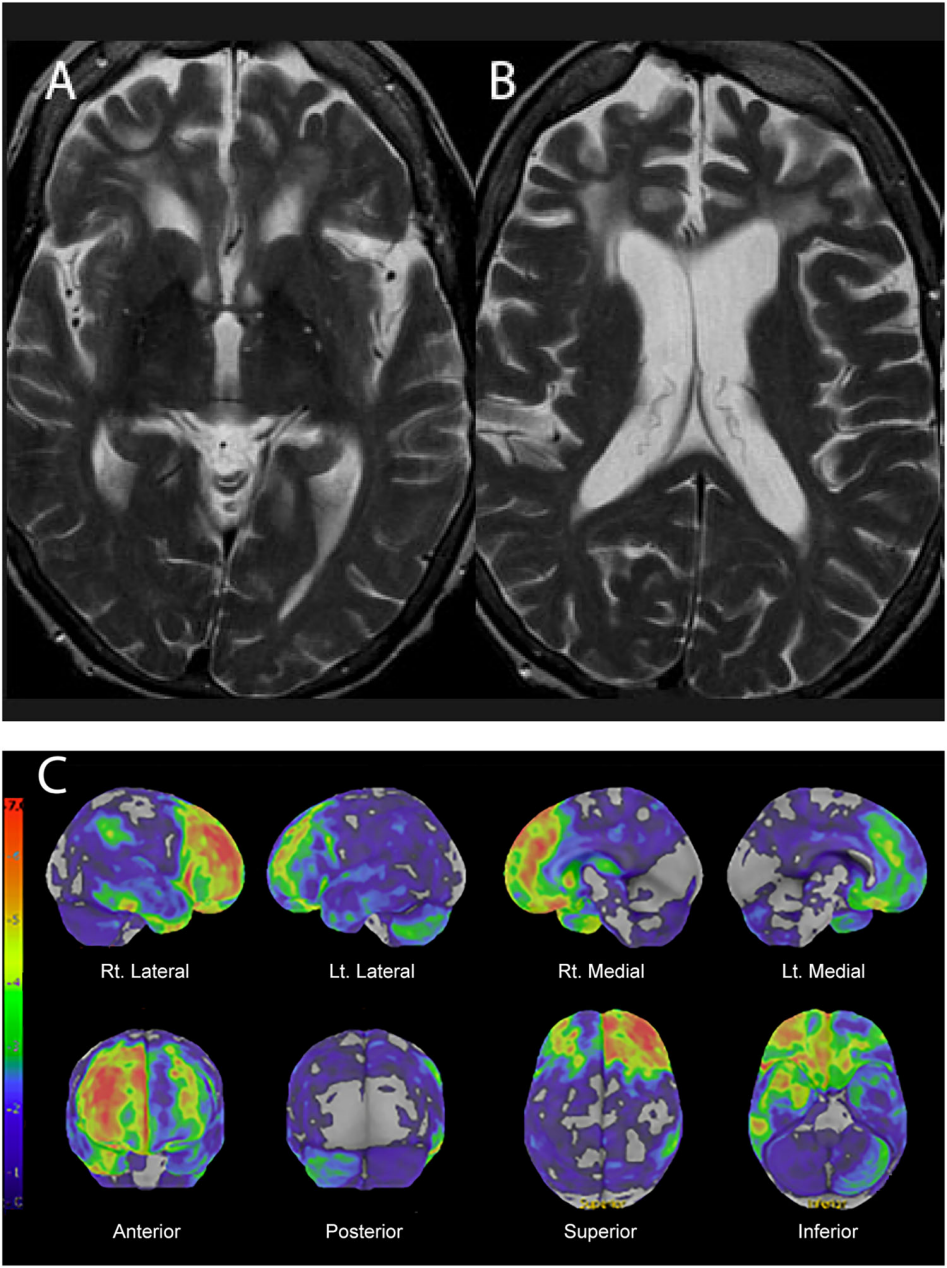
63-year-old woman with cognitive decline and unusual aggressive behavior. T2-weighted axial image at the basal ganglia (**A**) and the lateral ventricle (**B**) shows frontal dominant cortical atrophy with a moderate amount of white matter ischemic changes. FDG-PET with 3D-SSP reconstruction (**C**) shows profound frontal hypometabolism, right greater than left, with preservation of the posterior cingulate and parietal lobe. The findings are consistent with behavioral variant FTD

Neuroradiologists need to be aware of heterogeneity within AD. For example, patients with posterior cortical atrophy (PCA), known as a visual variant of AD, may present with visual symptoms, such as difficulty judging distances, and impairments in visual processing, such as bumping into doors while walking. They often present to ophthalmologists before seeking care from cognitive neurologists. Brain MRI shows profound occipital and posterior parietal atrophy while preserving frontal lobes (Fig. 2). Other AD variants include behavioral variants of AD, clinically resembling FTD (frontotemporal dementia).

### DLB (dementia with Lewy body)

**Fig. 3:**
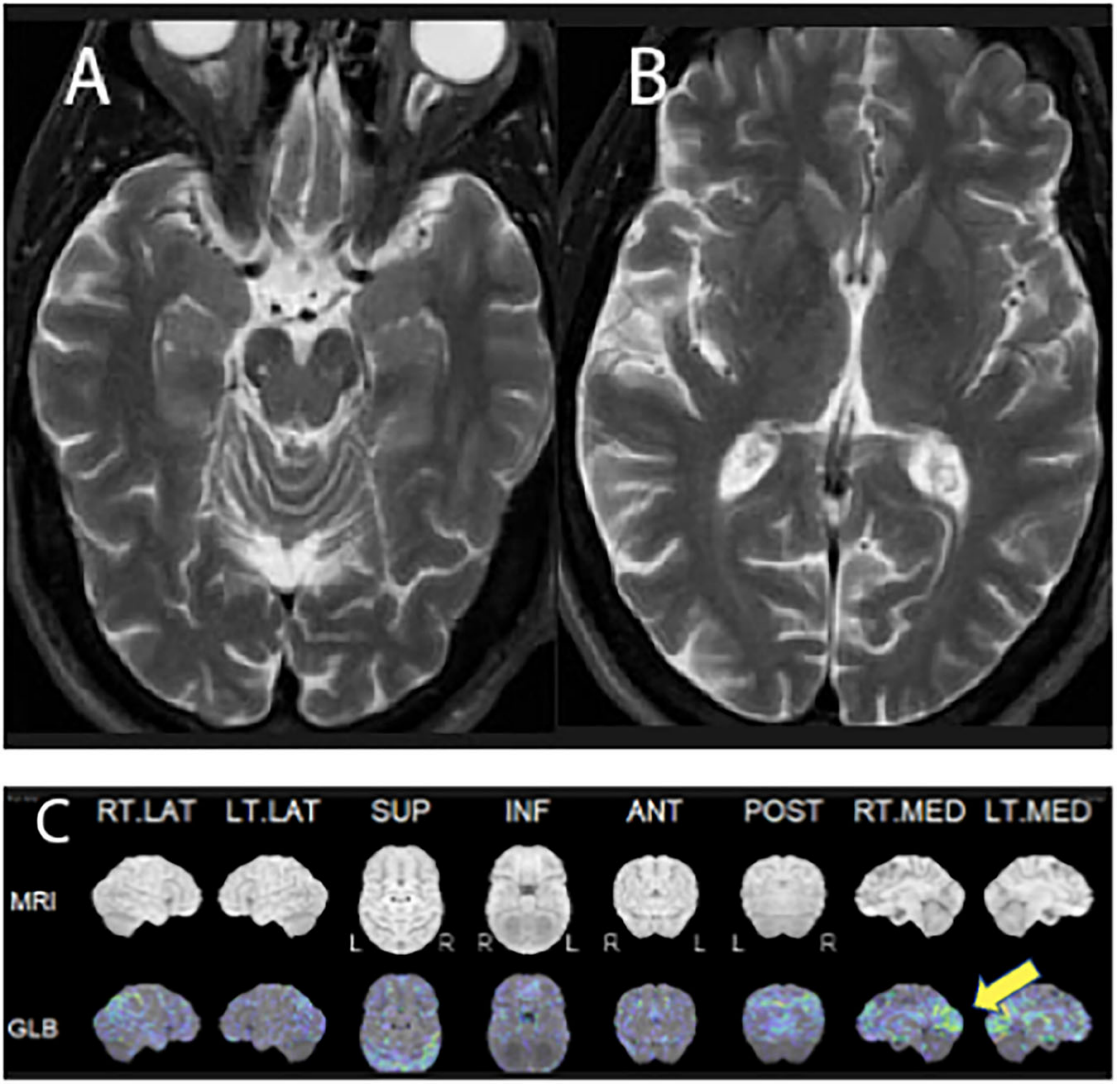
70-year-old male presents with a 1-year history of cognitive dysfunction, loss of executive function, diplopia, brain fog, short-term memory loss, disorientation, and REM sleep behavioral disturbance. T2-weighted axial image of the hippocampus (**A**) and the basal ganglia (**B**) demonstrates normal hippocampal volume; temporal and frontal lobes are unremarkable. FDG-PET with 3D-SSP reconstruction (**C**) shows profound occipital hypometabolism, right greater than left, but the posterior cingulate is preserved. The findings are consistent with dementia with Lewy body (DLB)

DLB affects more than 1 million patients in the US, with a slight male predominance. Symptoms related to DLB may include visual or auditory hallucinations and loss of cognitive ability, though memory may be preserved at least in the beginning. Patients often experience movement symptoms, such as rigidity, slowness, tremors, balance problems, and REM sleep behavior disorders. In most DLB cases, cognitive symptoms precede the motor signs, particularly visual hallucinations or diminished responsiveness. DLB is caused by the accumulation of alpha-synuclein. Brain MRI does not show a specific pattern of atrophy and often cannot differentiate from early AD (Fig. 3A, B). FDG-PET scan shows occipital hypometabolism with preservation or less involvement of posterior cingulate metabolism (cingulate island sign) (Fig. 3C), as well as hypometabolism in the substantia nigra and thalamus, allowing the supportive diagnosis [34]. Even though DLB is alpha-synucleinopathy, 11C-PIB amyloid PET scan shows diffuse accumulation of amyloid [35].

### FTLD (frontotemporal lobar degeneration)

FTLD refers to a group of diverse neurodegenerative disorders, and the classification of FTLD is refined either by clinical manifestation or underlying proteinopathy. Clinically, FTLD is a pathological condition manifest in FTD (frontotemporal dementia). FTD is less common and often affects younger patients than AD. Clinically, there are three types of FTD: (A) Behavioral variant of FTD (bvFTD), (B) PPA: A language-dominant FTD, and (C) FTD with atypical Parkinsonian syndrome.

(A) Behavioral variant of FTD (bvFTD) is a common subtype of FTD and includes Pick’s disease that is associated with neuropathological findings of tauopathy with Pick bodies. bvFTD manifests as a lack of ability to plan or execute tasks, impulsive behaviors, or a lack of interest in people or activity. Memory, however, tends to be preserved until late. Brain MR shows profound frontal lobe atrophy, affecting the lateral orbitofrontal gyrus bilaterally, though temporal lobe atrophy is mild (Fig. 2A, B). FDG-PET shows profound frontal lobe metabolic reduction, which is often asymmetric. This is frequently associated with mild metabolic reduction of the cerebellum, contralateral to the severely affected frontal lobe, suggestive of crossed cerebellar diaschisis (Fig. 2C).

(B) Primary progressive aphasia (PPA): a language-dominant FTD. PPA has three types: semantic PPA, nonfluent PPA (or agrammatic PPA), and logopenic PPA. Patients with semantic PPA struggle to name objects or understand the meaning of a word or a conversation. Underlying proteinopathy is primarily TDP-43 (Transactive Response DNA-Binding Protein of 43 kDa) as well as tauopathy. MR Imaging findings of semantic PPA show profound anterior temporal lobe atrophy (left more affected than right) with relative preservation of frontal and posterior temporal lobes. Nonfluent PPA manifests as effortful halting speech or apraxia of speech. Pathology often involves tauopathy, less frequently TDP-43. Brain MRI shows atrophy involving the left posterior frontal and insular atrophy. Semantic and nonfluent PPA will eventually progress to FTD. Logogenic PPA exhibits difficulty naming and repeating sentences and is not infrequently associated with cognitive and behavioral symptoms. Responsible proteinopathy are amyloid and tau. MRI and FDG-PET show posterior temporal and parietal atrophy and hypometabolism that resemble AD, and it is considered as a variant of AD, rather than a subtypes of FTLD. It is important to note that PPA is a clinical manifestation, not a neuropathological diagnosis. Accordingly, neuroradiologists need to categorize PPA into a subtype, when possible, to supplement clinical diagnosis.

(C) FTD with atypical Parkinsonian syndrome. In addition to bvFTD and PPA, progressive supranuclear palsy (PSP) (primarily semantic and nonfluent variants of PPS), corticobasal degeneration (CBD), and multiple system atrophy (MSA) are often classified into FTLD. The responsible proteinopathy is Tau pathology. PSP and CBD will be discussed later in this chapter.

#### LATE (limbic-predominant age-related TDP43 encephalopathy)

**Fig. 4:**
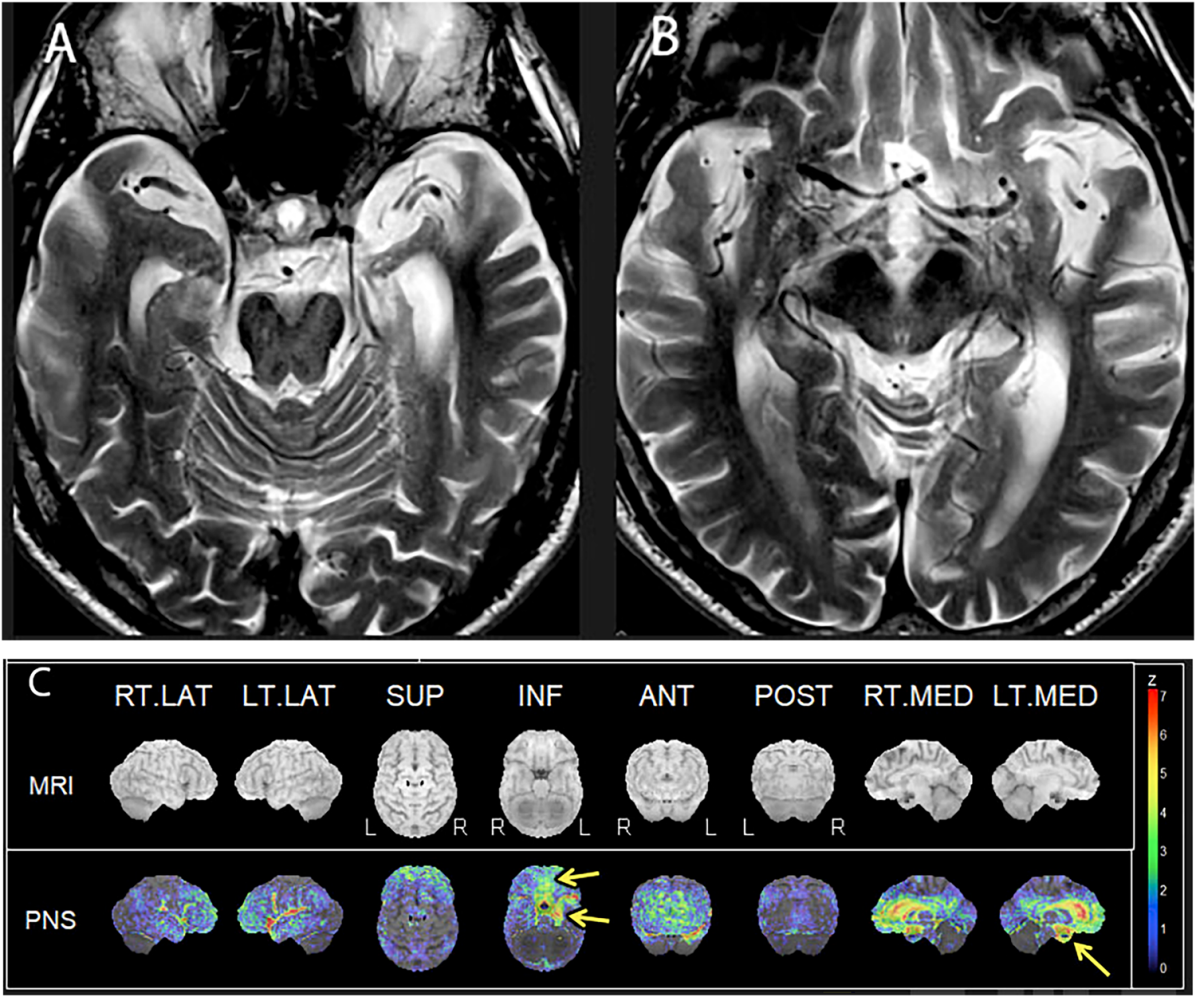
83-year-old male depressed since COVID, with a history of hypertension, hyperlipidemia, memory loss, word-finding difficulty, but still uses his cell phone. T2-weighted axial images of the hippocampus (**A**) and inferior temporal lobe (**B**) show severe atrophy of the hippocampus and amygdala, left greater than right. Furthermore, there are prominent sulci over the orbitofrontal cortex bilaterally. FDG-PET (**C**) with 3D-SSP reconstruction shows severe hypometabolism in the hippocampus and medial temporal lobes, left greater than right, and metabolic reduction in the medial orbitofrontal gyri (yellow arrows). The findings are consistent with LATE (limbic-predominant age-related TDP-43 encephalopathy)

A recently recognized entity called LATE highlights the complexity of neurodegenerative disease with overlapping clinical and imaging features. Autopsy study shows LATE neuropathologic changes are present in 20% of community-dwelling elderly patients. They present with amnestic cognitive impairment, mimicking AD. However, the underlying proteinopathy is TDP-43, primarily affecting the limbic structures commonly observed in patients past 80 years, so-called extreme old age [27]. The anatomical structures affected by LATE are the amygdala, hippocampus, and middle frontal gyrus in pathology (Fig. 4A, B). When you observe profound mesial temporal atrophy in amnestic extreme elderly patients, there is a great chance the patient has LATE. FDG-PET/CT shows profound hippocampal metabolic reduction in the medial temporal lobe, more than that observed in AD, and also associated with orbitofrontal gyrus [26] (Fig. 4C). The diagnosis of LATE is critically important for patients and family members as the progression is slower than that of AD. However, mixed pathology, particularly a combination of LATE and AD, is commonly seen in very elderly patients.

#### Movement disorders with cognitive impairment

Certain movement disorders also present with cognitive impairment. Understanding these conditions is essential for neuroradiologists as brain MR may suggest specific diagnoses in the appropriate clinical setting. These include, but are not limited to, Parkinson disease with dementia (PDD), PSP, CBD, and MSA.

#### PDD (Parkinson disease with dementia)

Parkinson disease is the second most common neurodegenerative disease affecting nearly one million people in the US, and more than 10 million people worldwide [36]. Men are 1.5 times more likely to have PD than women. Up to 50–70% of patients with PD will eventually develop dementia. The clinical distinction between PDD and DLB depends on the onset of first symptom, movement vs cognitive impairment, respectively. The DLB consortium supported a 1-year rule to differentiate DLB from PDD  [37]. Thus, if patients have PD for 1 year or longer before cognitive impairment, the diagnosis is likely PDD. If shorter, then DLB.  Both PD and DLB fall under the group of α-synucleinopathies.  In PD, there is an increase in iron deposition in the substantia nigra, the dorsolateral putamen and globus pallidus. Investigation of susceptibility-weighted MR imaging revealed the loss of high signal of nigrosome 1 in the substantia nigra, called the Swallow Tail sign, corresponding to a loss of dopaminergic neuron on the Dopaminergic receptor SPECT/CT scan (DaT scan) [38–41]. Normal high signal of nigrosome 1 excludes PD or Parkinson syndrome. This brings some excitement among neuroradiologists, though inter-observer variability remains a concern. QSM allows quantification of the amount of iron deposition, which may correlate with disease severity [42, 43].

#### PSP (progressive supranuclear palsy)

**Fig. 5:**
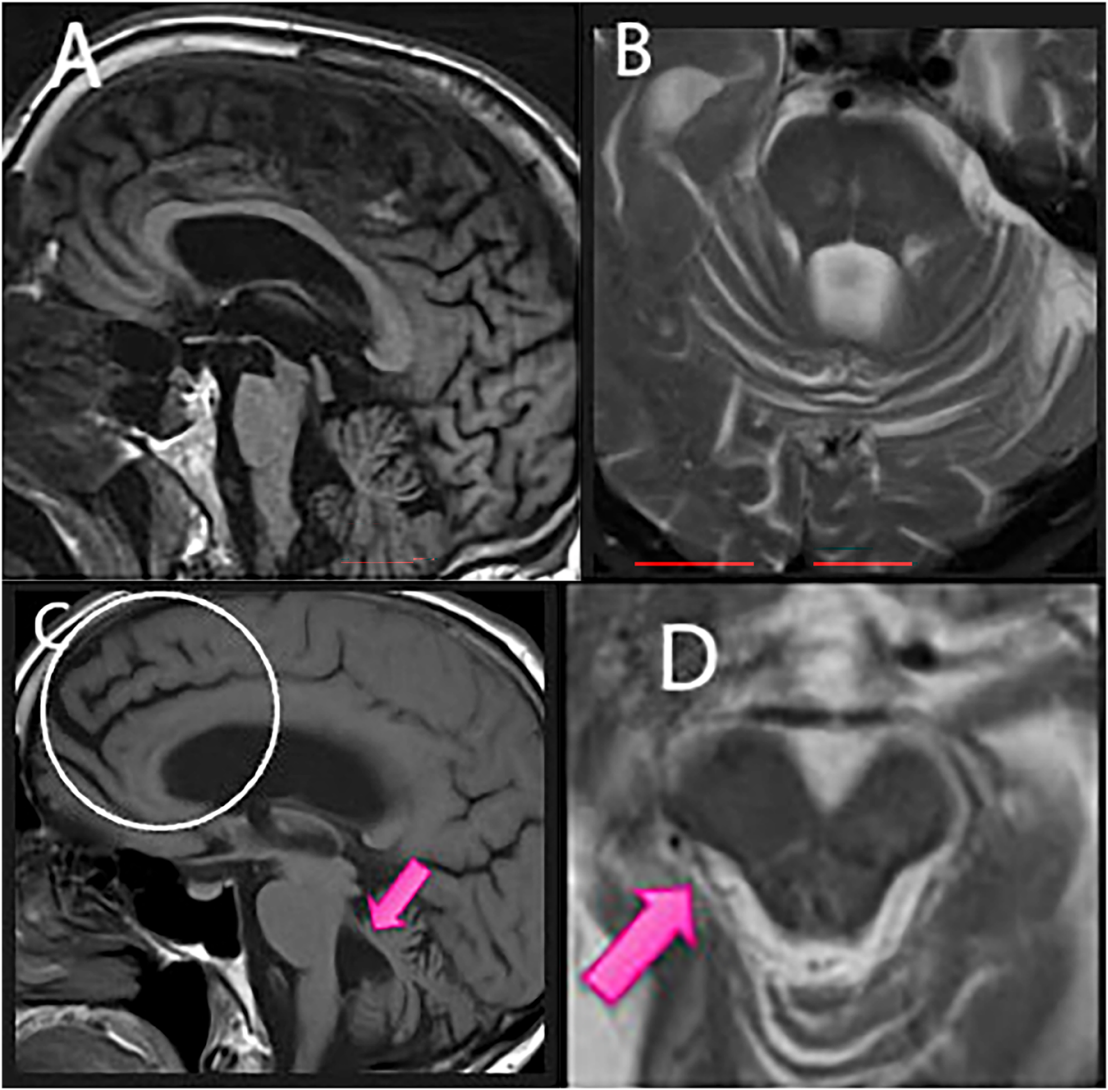
81-year-old male with atypical PD, tremor, loss of balance, dementia and vertical gaze difficulty. T1-weighted sagittal image (**A**) shows profound midbrain tegmental atrophy, manifesting a hummingbird sign. T2-weighted axial (**B**) image and T1-weighted sagittal image off-midline (**C**) show atrophy in the bilateral superior cerebellar peduncle and frontal dominant volume loss (circle). A magnified T2-weighted axial image (**D**) shows concavity along the posterior lateral midbrain (pink arrow), consistent with the morning glory sign. The findings are consistent with progressive supranuclear palsy (PSP)

PSP is a rare progressive NDD with movement disorders. PSP is considered a part of atypical PD (or so-called Parkinson-plus syndrome). Clinical presentation may include loss of balance, difficulty swallowing, slurred speech, and problems with an upper gaze. Brain MR findings of patients with PSP include atrophy in the midbrain tegmentum (hummingbird sign) (Fig. 5A), frontal lobe, superior and middle cerebellar peduncles, and globus pallidus (Fig. 5B, C). Another sign of concavity along the posterior lateral midbrain (morning glory sign) (Fig. 5D) is considered non-specific for PSP diagnosis. As a hummingbird sign can be subjective, several objective measurements have been proposed, including measurement of sagittal midbrain areas [44], as well as ventral tegmental midbrain distance (< 9.35 mm) and the ratio with that of pons (< 0.52) [45]. The ratio of the area of the midbrain and the area of the pons [46] can be useful for the differentiation of PSP from MSA [47]. Midbrain atrophy is also seen in the setting of idiopathic normal pressure hydrocephalus, one of the false positives for small midbrain [48]. Midline T1-weighted sagittal images are essential to make such a determination.

### CBD (corticobasal degeneration)

**Fig. 6:**
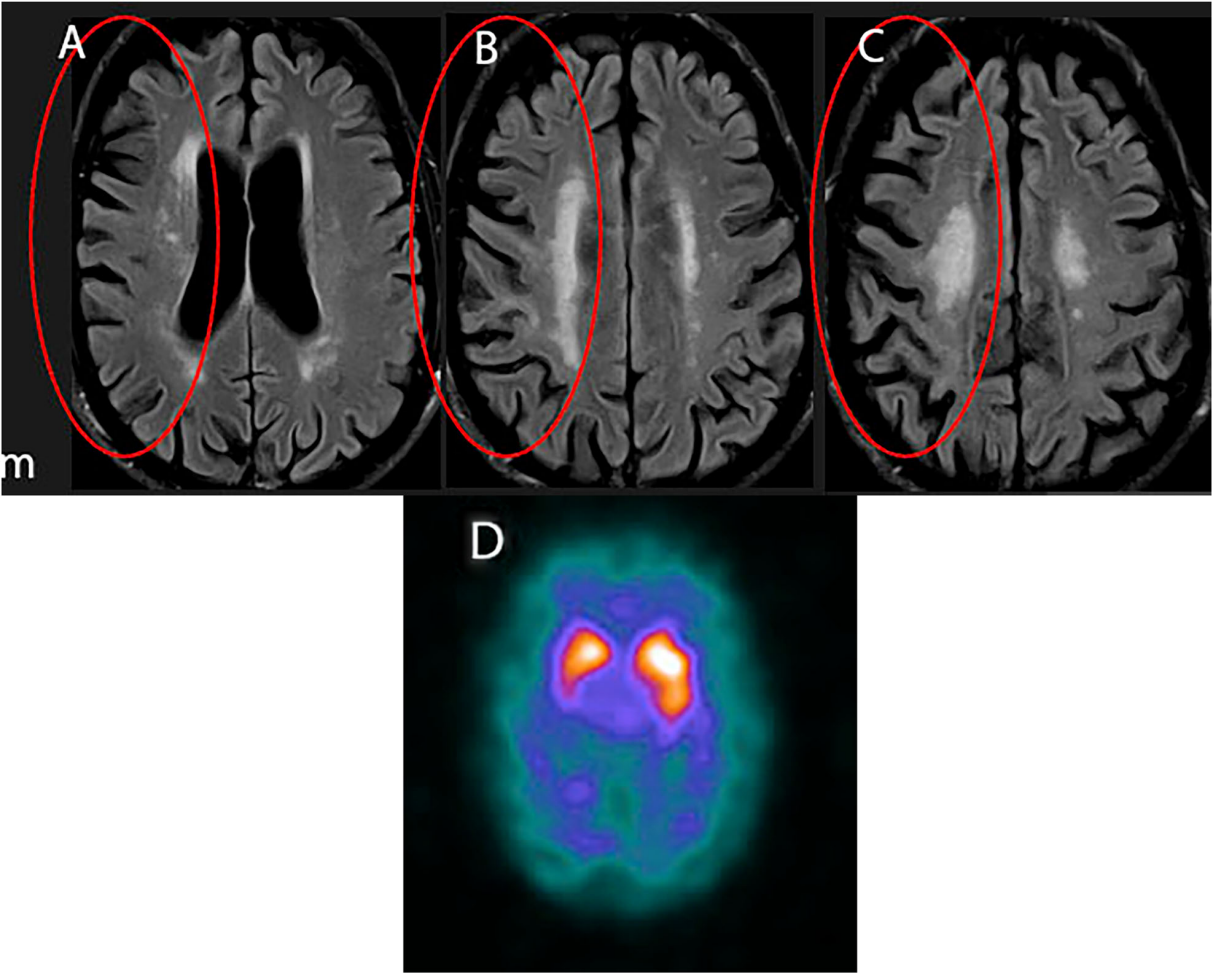
74-year-old male with tremor, rigidity, difficulty in using the left upper extremity, developed progressive nonfluent speech. MOCA reveals 18/30, unresponsive to levodopa. Executive function is preserved, and no personality changes are present. FLAIR axial images of the lateral ventricles (**A**), coronal radiata (**B**), and central semiovale (**C**) show profound asymmetric cortical volume loss over the right frontal and parietal cortex with asymmetric white matter T2 FLAIR hyperintensity, more in the right cerebral hemisphere. Dopaminergic receptor SPECT image (**D**) demonstrates asymmetric tracer reduction in the left striatum. The findings are consistent with corticobasal degeneration (CBD)

CBD is a slowly progressive disorder characterized by apraxia, dystonia, akinesia, rigidity, and postural instability. Classic symptoms of CBD include the so-called alien limb: a part of the limb is felt as if in someone else’s control. Brain MRI shows asymmetric perirolandic cortical atrophy contralateral to the side of the alien limb. In addition, T2 FLAIR hyperintensity is noted in the subcortical white matter of the affected side (Fig. 6A–C). FDG-PET shows hypometabolism in the perirolandic areas. Similar to PSP, CBD also shows midbrain tegmentum atrophy. PSP does not show subcortical white matter signal abnormality, however. CBD is caused by hyperphosphorylated 4-repeat tau protein aggregation, which is different from tau protein for AD (3-repeat and 4-repeat tau protein). Both CBD and PSP have degeneration of substantia nigra and locus coeruleus on pathology [49], though more severely affected on PSP. Dopaminergic receptor SPECT/CT scan (DaT scan) shows depletion of dopaminergic neuron activity (Fig. 5D).

### MSA (multisystem atrophy)

MSA is a rare condition affecting balance, motor, and autonomic functions. Although MSA is classified as MSA-c (cerebellar type) and MSA-p (Parkinson variant), MSA patients have similar clinical manifestations, with some symptoms being more dominant than others. The imaging appearance of MSA may depend on the predominant clinical manifestation. For example, MSA-c is associated with ataxia and cerebellar symptoms. Brain MR shows the so-called hot cross bun sign, substantial transverse pontine fiber loss with preservation of corticospinal tract, and atrophy of the cerebellum and middle cerebellar peduncle. Patients with MSA-p may exhibit predominant extrapyramidal motor dysfunction, similar to PD, such as rigidity, slow movement, postural instability, and increased iron deposition (gradient susceptibility) in the dorsolateral putamen as well as putaminal atrophy on MR and hypometabolism on FDG-PET. Although the clinical manifestation of MSA-p is similar to that of PD patients, MSA-p symptoms do not respond to levodopa therapy.

#### Co-pathology of neurodegenerative disease

Two or more pathologies often co-exist in patients with dementia. The most common combination is AD plus vascular dementia. However, various other combinations, such as AD plus DLB, AD plus FTD, AD plus CBD, and AD plus PD, can be seen in the same subject. In addition, increasingly recognized amnestic dementia, LATE, is seen in elderly patients over age 80, who frequently have amyloid positivity without a clear signature of metabolic reduction of AD phenotype. Furthermore, NPH plus AD is also increasingly recognized co-pathology in elderly patients. In those patients, even though patients may respond to high-volume CSF tap or lumbar shunt, cognitive symptoms may not improve if co-existing AD is present. Given the complexity and heterogeneity of the neurodegenerative disease, patients may not fit into one specific disease entity. Neuroradiologists need to understand which phenotype can explain a patient’s clinical symptoms so that proper management and consultation can be performed with patients and their family members.

In summary, neurodegenerative disease (NDD) is a complex, heterogeneous disorder. Understanding clinical manifestations and dominant symptoms is essential to guiding the differential diagnosis of NDD. A multidisciplinary team comprising cognitive neurologists, psychiatrists, neuroradiologists, and molecular imaging specialists is critical to providing pertinent information to patients and their family members.

## Abbreviation

AD: Alzheimer disease
CBD: Corticobasal degeneration
DLB: Dementia with Lewy body
FTLD: Frontotemporal lobar degeneration
LATE: Limbic-predominant age-related TDP43 encephalopathy
MSA: Multisystem atrophy
PD: Parkinson disease
PSP: Progressive supranuclear palsy
TDP 43: Transactive response DNA-binding protein of 43 kDa
